# Gout in pregnancy: Obstetric and neonatal outcomes

**DOI:** 10.1002/ijgo.16025

**Published:** 2024-11-19

**Authors:** Sam Amar, Ahmad Badeghiesh, Haitham Baghlaf, Michael H. Dahan

**Affiliations:** ^1^ Faculty of Medicine and Health Sciences McGill University Montreal Quebec Canada; ^2^ Department of Obstetrics and Gynecology King Abdulaziz University Rabigh Saudi Arabia; ^3^ Department of Obstetrics and Gynecology University of Tabuk Tabuk Saudi Arabia; ^4^ Department of Obstetrics and Gynecology McGill University Montreal Quebec Canada

**Keywords:** congenital anomalies, gestational diabetes mellitus, gout, maternal‐fetal medicine, pregnancy, pulmonary embolism

## Abstract

**Objective:**

The pregnancy, delivery, and neonatal outcomes of pregnancies complicated by gout have yet to be evaluated in a population‐based study. We sought to evaluate the obstetric and neonatal outcomes in pregnant patients with gout using a national population database.

**Methods:**

This is a retrospective population‐based cohort study utilizing the Healthcare Cost and Utilization Project, Nationwide Inpatient Sample (HCUP‐NIS). All women who delivered or had a maternal death in the USA (2004–2014) were included in the study. Pregnancy, delivery, and neonatal outcomes were compared between women with an ICD‐9 diagnosis of gout to those without.

**Results:**

Overall, 9 096 788 women met the inclusion criteria. Of these, 168 women (1.8/100000) had gout. Patients with gout, compared to those without, were more likely to be older and obese and to have chronic hypertension, pregestational diabetes mellitus, and thyroid disease. Pregnant women with gout were more likely to develop gestational diabetes mellitus (aOR 1.78 [95% CI 1.17–2.72], *P* = 0.008), to require operative vaginal delivery (aOR 3.26 [95% CI 2.03–5.22], *P* = 0.0001), and to experience venous thromboembolism (aOR 8.47 [95% CI 2.06–34.82], *P* = 0.003) compared to pregnant patients without gout. Patients with gout were more likely to deliver a neonate with congenital anomalies compared to those without gout (aOR 3.38 [95% CI 1.24–9.20], *P* = 0.02).

**Conclusion:**

Gout in pregnancy, or pregnancies complicated by a history of gout, are associated with increased risk of gestational diabetes mellitus, pulmonary embolism, and neonatal anomalies.

## Synopsis

Gout in pregnancy is associated with an increased risk of gestational diabetes mellitus, pulmonary embolism, and neonatal congenital anomalies.

## 1 INTRODUCTION

Gout is a common type of arthritis that presents as episodic flares of painful joint inflammation.^1^ The arthritis is typically monoarticular and affects a lower limb joint, most commonly the first metatarsophalangeal joint.^2^ Risk factors for gout include genetic and dietary factors, in addition to medical comorbidities such as insulin resistance.^2^ These risk factors contribute to the development of hyperuricemia, which enables monosodium urate crystals to form in joints and other soft tissues. The joint pain and inflammation in gout are due to the innate immune system's response to these crystals.^3^ In addition to causing gout, hyperuricemia is an independent risk factor for myocardial infarction and stroke.^4^, ^5^ As such, high uric acid levels might affect the vasculature and produce a hypercoagulable state. Gout's prevalence is increasing worldwide.^2^ Although the general incidence of gout is between 0.6 and 2.9 per 1000 person‐years,^3^ its incidence in women of reproductive age is significantly lower at 1.6 per 10 000 person‐years.^6^ This discrepancy might be explained by the fact that estrogen promotes renal excretion of uric acid.^7^ As such, increased estrogen levels during pregnancy might protect women from acute gout flares.

Many patients with gout suffer from various comorbidities, such as diabetes and hypertension.^8^ Pregnant women with these chronic conditions are at increased risk of adverse obstetric and neonatal outcomes, such as preterm delivery, fetal growth restriction, and preeclampsia.^9^, ^10^ However, there is a paucity of data about whether the presence of gout itself further increases the risks of adverse pregnancy and delivery outcomes when controlling for the rates of these inherent risk factors. Moreover, the current literature about gout in pregnancy is mainly limited to case reports or small retrospective studies.^7^, ^11^, ^12^, ^13^ These studies suggest that gout in pregnancy might be associated with gestational diabetes, cesarean delivery, and preterm birth, but a larger study is warranted to confirm these effects. Additionally, given the adverse cardiovascular and cerebrovascular effects associated with hyperuricemia,^4^, ^5^ it is of great importance to determine whether pregnancy, a state of vascular remodeling and hypercoagulability, is affected by high uric acid levels. Therefore, we sought to evaluate the obstetric and neonatal outcomes in pregnant patients with gout using a national population database.

## 2 MATERIALS AND METHODS

### 2.1 Study design

This was a retrospective population‐based cohort study, using the Healthcare Cost and Utilization Project Nationwide Inpatient Sample (HCUP‐NIS) database between 2004 and 2014,^14^ inclusively. The HCUP‐NIS is the largest inpatient sample database in the USA and is comprised of hospital inpatient stays submitted by hospitals throughout the country. Each year, the database provides information relating to 7 million inpatient stays, including patient characteristics, diagnoses, and procedures. The data represent 20% of admissions to US hospitals across 48 states and the District of Columbia.

Collected data included demographic and obstetric parameters, labor, and short‐term maternal and neonatal outcomes up to the time of discharge. Demographic parameters included maternal age, race, income, medical insurance type, previous cesarean delivery, and multiple pregnancies. Maternal outcome data included pregnancy‐induced hypertension, eclampsia, preeclampsia, gestational diabetes mellitus (GDM), placental abruption, placenta previa, preterm premature rupture of membranes (PPROM), preterm delivery (<37 weeks), chorioamnionitis, operative vaginal delivery, cesarean delivery (CD), postpartum hemorrhage (PPH), wound complications, peripartum hysterectomy, maternal death, maternal infection, need for blood transfusion, deep vein thrombosis (DVT), pulmonary embolism (PE), venous thromboembolism (VTE), and disseminated intravascular coagulation (DIC). Neonatal outcomes examined included being small‐for‐gestational‐age (SGA) neonates, defined as birthweight below the tenth percentile; intra‐uterine fetal death (IUFD); and congenital anomalies.

### 2.2 Study participants

The pregnant subjects included had to have either a delivery or a maternal death, such that they would be included only once per pregnancy in the database. Our cohort included only a possibly viable pregnancy at 24 weeks or above and did not include earlier miscarriages. The cohort was divided into two groups according to gout diagnosis: women diagnosed with gout and the rest of the population, which acted as a comparison group. Patients' status was categorized based on an ICD‐9 diagnosis of gout, which was based on the code 274.9. Remaining subjects acted as controls. The data was limited up until 2014 because in 2015 the HCUP database started coding with ICD‐10 codes, which are not compatible with ICD‐9 codes. This study used exclusively publicly accessible, anonymized data; therefore, according to articles 2.2 and 2.4 of the Tri‐Council Policy Statement (2010),^15^ institutional review board approval and informed consent from participants were not required.

### 2.3 Statistical analysis

An initial analysis was performed to identify the incidence of women diagnosed with gout over the entire study duration. Subsequently, *χ* ^2^‐tests were used to compare the baseline characteristics between women with a gout diagnosis and those without. Logistic regression analyses were then performed to assess the unadjusted and adjusted effect of a gout diagnosis on maternal and neonatal outcomes through the estimation of odds ratio (OR) and 95% confidence intervals (CI). The regression models were adjusted for the potential confounding effects of maternal demographics, pre‐existing clinical characteristics, and concurrently occurring conditions where *P*‐values of <0.05 were identified in the *χ* ^2^ analysis. All analyses were performed using SPSS 23.0 (IBM Corporation, Chicago, USA).

Per convention using the HCUP data, any group with less than 11 occurrences were represented as ≤10 to prevent loss of anonymity, in the tables, with the exception of 0 cases in which there was no risk to anonymity.^14^

## 3 RESULTS

Of the 9 096 788 patients that met the inclusion criteria, 168 had a diagnosis of gout (1.8/100000). The prevalence of gout in pregnancy increased over the study period from 0.68 to 3.67 per 100 000 births (*P* < 0.0001). Table 1 presents the demographic and baseline characteristics of women with and without gout. Patients with gout, compared to those without, were more likely to be older (*P* = 0.0001) and to use Medicare insurance (*P* = 0.0001). Women with gout were more likely to be obese and have chronic hypertension, pregestational diabetes mellitus, and thyroid disease compared to women without gout (*P* = 0.0001 for all). Patients with gout were more likely to have had a previous cesarean delivery compared to those without (*P* = 0.03). Patients with gout had higher rates of illicit drug use (*P* = 0.03) relative to women without gout.

**TABLE 1:** Maternal characteristics.

Characteristics	Gout, *N* = 168	No gout, *N* = 9 096 620	*P*‐value
Age (years)			**0.0001**
<25	23 (13.7)	3 455 832 (38.0)	
25–34	70 (41.7)	4 299 833 (47.3)	
≥35	75 (44.6)	1 340 945 (14.7)	
Race			0.67
White	80 (47.6)	4 482 579 (49.3)	
Black	37 (22.0)	1 649 347 (18.1)	
Hispanic	27 (16.1)	2 029 301 (22.3)	
Asian and Pacific Islander	16 (9.5)	441 799 (4.9)	
Native American	2 (1.2)	64 146 (0.7)	
Other	5 (3.0)	365 655 (4.0)	
Income quartiles			0.66
<$39 000	34 (20.2)	2 218 106 (24.4)	
$39000–47 999	65 (38.7)	3 080 065 (33.9)	
$48000–62 999	43 (25.6)	2 416 093 (26.6)	
$63 000 or more	26 (15.5)	1 382 304 (15.2)	
Plan type			**0.0001**
Medicare	13 (7.7)	56 590 (0.6)	
Medicaid	61 (36.3)	3 882 715 (42.7)	
Private including HMO	90 (53.6)	4 606 883 (50.6)	
Self‐pay	0 (0)	288 436 (3.2)	
No charge	1 (0.6)	17 061 (0.2)	
Other	3 (1.8)	244 935 (2.7)	
Obesity	50 (29.8)	324 126 (3.6)	**0.0001**
Previous cesarean section	36 (21.4)	1 452 454 (16.0)	**0.03**
Smoking during pregnancy	11 (6.5)	443 579 (4.9)	0.20
Chronic hypertension	40 (23.8)	165 190 (1.8)	**0.0001**
Pregestational diabetes mellitus	24 (14.3)	86 591 (1.0)	**0.0001**
Drug use	6 (3.6)	125 613 (1.4)	**0.03**
Multiple gestation	2 (1.2)	137 301 (1.5)	0.53
Thyroid disease	16 (9.5)	223 262 (2.5)	**0.0001**
Human immunodeficiency virus infection	0 (0)	2079 (0)	0.96
In‐vitro fertilization	1 (0)	0 (0)	0.19

*Note*: Bold values are statistically significant (*p* < 0.05).

Table 2 demonstrates the association between gout and pregnancy and delivery outcomes after adjusting for potential confounders (listed under the table). Patients with gout, compared to those without, had an increased likelihood of developing gestational diabetes mellitus (adjusted odds ratio [aOR] 1.78 [95% CI 1.17–2.72], *P* = 0.008). Women with gout were also more likely to experience an operative vaginal delivery (aOR 3.26 [95% CI 2.03–5.22], *P* = 0.0001) and less likely to have a spontaneous vaginal delivery (aOR 0.64 [95% CI 0.46–0.89], *P* = 0.008) compared to patients with no gout. Gout in pregnancy was associated with an increased likelihood of requiring a blood transfusion compared to not having gout [aOR 2.49 (95% CI 1.15–5.39), *P* = 0.02]. Pulmonary embolism (aOR 8.90 [95% CI 1.20–65.97], *P* = 0.03) and venous thromboembolism (aOR 8.47 [95% CI 2.06–34.82], *P* = 0.003) were more likely to occur in pregnant patients with gout compared to those without gout. In univariate analysis, women with gout were more likely to experience gestational hypertension, eclampsia, preeclampsia superimposed on chronic hypertension, preterm delivery, and cesarean section, but these variables did not retain their significance in the multivariate analysis.

**TABLE 2:** Pregnancy and delivery outcomes.

Outcomes	Gout (%)	No gout (%)	Crude OR (95% CI)	Adjusted OR (95% CI)	Adjusted *P*‐value
Pregnancy outcomes^a^					
Pregnancy‐induced hypertension	29 (17.3)	673 720 (7.4)	2.61 (1.75–3.89)	1.14 (0.74–1.75)	0.56
Gestational hypertension	≤10	301 598 (3.3)	1.65 (0.84–3.23)	1.37 (0.69–2.69)	0.37
Preeclampsia	≤10	327 381 (3.6)	1.51 (0.78–2.97)	1.01 (0.51–2.01)	0.97
Eclampsia	≤10	6943 (0.1)	7.84 (1.10–55.99)	6.85 (0.95–49.16)	0.056
Preeclampsia and eclampsia superimposed hypertension	11 (6.5)	47 354 (0.5)	13.39 (7.27–24.68)	1.01 (0.50–2.05)	0.97
Gestational diabetes mellitus	30 (17.9)	523 162 (5.8)	3.56 (2.40–5.29)	1.78 (1.17–2.72)	**0.008**
Placenta previa	≤10	49 981 (0.5)	1.08 (0.15–7.74)	0.76 (0.11–5.41)	0.78
Delivery outcomes^b^					
Preterm premature rupture of membranes	≤10	103 617 (1.1)	0.52 (0.07–3.71)	0.38 (0.05–2.70)	0.33
Preterm delivery	21 (12.5)	653 874 (7.2)	1.85 (1.17–2.91)	1.13 (0.70–1.81)	0.62
Abruptio placenta	≤10	97 478 (1.1)	0.55 (0.08–3.95)	0.40 (0.06–2.90)	0.37
Chorioamnionitis	≤10	165 324 (1.8)	2.00 (0.89–4.52)	2.01 (0.89–4.54)	0.09
Operative vaginal delivery	20 (11.9)	489 381 (5.4)	2.38 (1.49–3.79)	3.26 (2.03–5.22)	**0.0001**
Cesarean delivery	85 (50.6)	2 939 833 (32.3)	2.15 (1.59–2.90)	1.07 (0.78–1.48)	0.66
Spontaneous vaginal delivery	63 (37.5)	5 667 406 (62.3)	0.36 (0.27–0.50)	0.64 (0.46–0.89)	**0.008**
Hysterectomy	0 (0)	7099 (0.1)	N/A	N/A	N/A
Postpartum hemorrhage	≤10	263 959 (2.9)	1.24 (0.55–2.80)	1.11 (0.49–2.50)	0.81
Wound complications	≤10	32 732 (0.4)	1.66 (0.23–11.84)	0.73 (0.10–5.22)	0.75
Maternal death	0 (0)	638 (0)	N/A	N/A	N/A
Transfusion	≤10	90 360 (1.0)	4.42 (2.07–9.43)	2.49 (1.15–5.39)	**0.02**
Others					
Maternal infection	≤10	199 261 (2.2)	1.94 (0.91–4.14)	1.85 (0.87–3.95)	0.11
Deep vein thrombosis	≤10	3831 (0.0)	14.21 (1.99–101.51)	6.96 (0.96–50.19)	0.054
Pulmonary embolism	≤10	1658 (0.0)	32.85 (4.60–234.69)	8.90 (1.20–65.97)	**0.03**
Venous thromboembolism	≤10	5308 (0.1)	20.63 (5.12–83.22)	8.47 (2.06–34.82)	**0.003**
Disseminated intravascular coagulation	0 (0)	18 244 (0.2)	N/A	N/A	N/A

*Note*: When no occurrences of a complication were noted for a group, the odds ratio (OR) cannot be calculated and is represented as N/A. Bold values are statistically significant (*p* < 0.05).

Abbreviations: CI, confidence interval. OR, odds ratio.

^a^ Pregnancy outcomes: Adjusted for age, plan type, drug use, chronic hypertension, obesity, thyroid disease, and pregestational diabetes mellitus.

^b^ Delivery outcomes: Adjusted for age, plan type, drug use, chronic hypertension, obesity, thyroid disease, pregestational diabetes mellitus and gestational diabetes mellitus.

Neonatal outcomes are presented in Table 3. After controlling for confounding factors, babies born to women with gout had a higher incidence of congenital anomalies compared to those born to women without gout (aOR 3.38 [95% CI 1.24–9.20], *P* = 0.02).

**TABLE 3:** Neonatal outcomes^a^.

Outcomes	Gout (%)	No gout (%)	Crude OR (95% CI)	Adjusted OR (95% CI)	Adjusted *P*‐value
Small for gestational age	≤10	198 066 (2.2)	1.10 (0.41–2.96)	0.75 (0.28–2.04)	0.57
Intrauterine fetal death	≤10	38 258 (0.4)	1.42 (0.20–10.12)	0.69 (0.10–4.96)	0.71
Congenital anomalies	≤10	38 240 (0.4)	5.78 (2.14–15.58)	3.38 (1.24–9.20)	**0.02**

*Note*: Bold values are statistically significant (*p* < 0.05).

Abbreviation: OR, odds ratio.

^a^ Adjusted for age, plan type, drug use, chronic HTN, obesity, thyroid disease, pregestational DM and gestational DM.

## 4 DISCUSSION

This is the largest retrospective study evaluating the pregnancy, delivery, and neonatal outcomes of patients with gout. Women who experienced gout were more likely to be older, to use Medicare, and to have had a cesarean section compared to women without gout. Patients with gout also had higher rates of comorbidities, such as obesity, chronic hypertension, pregestational diabetes mellitus, and thyroid disease, and were more likely to be drug users. Patients with gout, compared to those without, were more likely to develop gestational diabetes mellitus and pulmonary embolism. They were also more likely to require an operative vaginal delivery and a blood transfusion and were less likely to have a spontaneous vaginal delivery. Neonates born to mothers with gout were more likely to have congenital anomalies. Our study is the first to demonstrate these findings.

Currently, there is limited data about the effects of gout on pregnancy. A literature review of 11 studies (nine case reports, one retrospective cohort study, and one prospective cohort study) observed that rates of gestational diabetes might be increased in patients with gout compared to those with no gout, which concurs with our results.^7^ This study also observed slightly elevated rates of preeclampsia in pregnancies complicated by gout compared to the general population. Furthermore, Chen et al. conducted a population‐based study of 473 529 pregnancies in Taiwan and observed that women with gout were more likely to develop preeclampsia, to require cesarean delivery, to have preterm delivery, and to deliver an infant of low birth weight who was small for gestational age.^16^ Women with gout in our univariate analysis were more likely to experience a cesarean section and preterm delivery compared to women without. However, none of the outcomes that Chen et al. found to be associated with gout in their study were significant in our multivariate analysis, when controlling for confounding effects. Furthermore, a case–control study of 215 patients observed that pregnancies complicated by preeclampsia and hyperuricemia had increased rates of preterm birth and smaller infants for gestational age compared to preeclampsia alone.^17^ In contrast, women with gout in our study were not more likely to have preeclampsia compared to women without.

A greater proportion of patients with gout in our study suffered from comorbid conditions compared to the rest of our pregnant population, such as pregestational diabetes mellitus, chronic hypertension, obesity, and thyroid disease. Additionally, the women with gout were older than those without. Likewise, gout in the general, nonpregnant population is associated with these same comorbidities.^2^ Various studies have indicated that diabetes and hypertension in pregnancy are associated with an array of adverse outcomes. Pregnancies complicated by diabetes mellitus have increased rates of congenital anomalies, fetal macrosomia or fetal growth restriction, cesarean delivery, gestational hypertension, and preterm birth.^18^, ^19^ Chronic hypertension has been associated with superimposed preeclampsia/eclampsia, fetal growth restriction, placental abruption, preterm birth, and cesarean sections.^20^ As patients get older, they have an increased risk of gestational diabetes, preeclampsia, placenta previa, low birth weight, preterm delivery, stillbirth, cesarean section, thrombosis, and postpartum hemorrhage.^21^ Many of these outcomes, such as eclampsia, superimposed preeclampsia, preterm delivery, and cesarean section requirement were associated with gout in our univariate analysis but not in our multivariate analysis. The increased rates of these pregnancy complications in women with gout are therefore most likely due to their comorbid conditions as opposed to the gout itself. However, our study suggests that women with gout have increased risk of gestational diabetes, operative vaginal delivery, and pulmonary embolism compared to women with the same comorbidities who do not have gout.

In our study, gout in pregnancy was associated with gestational diabetes mellitus after controlling for other comorbidities. Similarly, the patient in the case report by van Veen et al. also developed GDM during her pregnancy complicated by a gout flare.^7^ A couple of studies have investigated the relationship between insulin and uric acid levels.^22^, ^23^ A population study of 14 664 Americans demonstrated that higher insulin levels were associated with hyperuricemia.^22^ Similarly, a cross‐sectional study of 36 healthy volunteers observed a linear association between insulin resistance (measured by steady state plasma glucose concentration) and increased uric acid levels.^23^ Both studies postulated that insulin resistance and hyperinsulinemia decrease renal uric acid clearance, resulting in hyperuricemia. As such, van Veen et al. hypothesized that the gout flare in their pregnant patient was provoked by gestational diabetes mellitus.^7^ The interplay between insulin physiology and hyperuricemia might also explain why women with gout in our study had a higher likelihood of experiencing GDM compared to women without gout.

We observed that patients with gout had a higher risk of pulmonary embolism compared to those without gout after controlling for possible confounders. Although our study is the first to document this finding in pregnancy, previous studies have found an association between gout in the general population and venous thromboembolism. A prospective study of 14 758 individuals observed that those who developed VTE had significantly higher uric acid levels than those without.^24^ Additionally, a prospective cohort study of 71 918 patients observed that those with gout had a higher likelihood of experiencing DVT compared to patients without.^25^ In our study, the association between gout and DVT in pregnancy was almost statistically significant and might have been significant with a larger sample size. Nonetheless, the increased likelihood of pulmonary embolism in our patients with gout might be explained by the fact that uric acid can induce vascular endothelial injury through oxidative stress and inflammation. This endothelial injury predisposes to thrombosis.^26^

### 4.1 Clinical implications

Our study is the first to document the association between gout in pregnancy and newborn congenital anomalies. As such, patients who experience gout during pregnancy, or are known to have a history of gout, should be counseled about this increased risk. Clinicians might consider the possibility of more detailed antenatal ultrasound scanning in this population. Additionally, given that gestational diabetes mellitus is more likely to develop in patients with gout compared to those without, pregnant patients with a history of gout should receive early screening for gestational diabetes mellitus. Alternatively, if a patient experiences a gout flare for the first time during pregnancy, they should be promptly screened for GDM even in the absence of other risk factors. Given the association between gout and adverse pregnancy and neonatal outcomes identified in this study, pregnant patients with gout should undergo consultation with maternal–fetal medicine or obstetric medicine to try and mitigate this risk.

### 4.2 Research implications

Given the increased rates of pulmonary embolism in pregnant patients with gout, future studies should investigate whether preventative measures could be used to decrease this risk. For example, researchers could evaluate if low molecular weight heparin is effective in preventing venous thromboembolism in patients with a history of gout or if screening for deep venous thrombosis in pregnant patients with gout is effective in preventing pulmonary embolism. Additionally, the increased risks of congenital anomalies in women with gout is possibly due to the medications used to prevent gout flares and should be studied in a database that permits linkage to medications used. The HCUP database does not permit such analyses.

### 4.3 Strengths and limitations

To our knowledge, this is the largest study to date about the impacts of gout on pregnancy outcomes. However, our study has several limitations. First, the multicenter retrospective study design prevented us from overseeing the data collection and limited the type of data we could evaluate. Next, some maternal characteristics were not recorded, such as thrombophilia status, autoimmune disease, and congenital heart disease, which might also affect perinatal outcomes in pregnancies complicated by gout. Additionally, we could not determine whether the patients in our study experienced gout attacks before or during their pregnancy. As such, we are unable to determine whether the significant outcomes in our study are associated with acute gout flares, with a history of gout, or both. Similarly, for gout attacks that occurred during pregnancy, we are unable to assess whether the attack occurred earlier or later in gestation. This limitation must be taken into account when considering the association between gout and congenital anomalies and the recommendation for GDM screening. Moreover, we did not have access to the patients' uric acid levels, which might have also influenced pregnancy outcomes.

In conclusion, our findings suggest that gout in pregnancy is associated with various adverse maternal outcomes, such as gestational diabetes mellitus, pulmonary embolism, and operative vaginal delivery. Pregnant patients with gout also have an increased risk of delivering a neonate with congenital anomalies. Patients with a history of gout, or those who experience a flare during pregnancy, should be counseled about these adverse outcomes and should be offered appropriate diagnostic testing.

## AUTHOR CONTRIBUTIONS

**Sam Amar:** Protocol and project development, data collection and management, data analysis, and manuscript writing and editing. **Ahmad Badeghiesh:** Protocol and project development, data collection and management, data analysis, and manuscript editing. **Haitham Baghlaf:** Protocol and project development, data collection and management, data analysis, and manuscript editing. **Michael H. Dahan:** Protocol and project development, data collection and management, data analysis, and manuscript writing and editing.

## FUNDING INFORMATION

No sources of funding to report.

## CONFLICT OF INTEREST STATEMENT

The authors declare no conflicts of interest.

## Data Availability

The data of the Healthcare Cost and Utilization Project Nationwide Inpatient Sample (HCUP‐NIS) database between 2004 and 2014 is publicly available in anonymized form. The data that support the findings of this study are openly available in the Healthcare Cost and Utilization Project Nationwide Inpatient Sample (HCUP‐NIS) at https://hcup‐us.ahrq.gov/nisoverview.jsp.

## References

[1] Narang RK , Dalbeth N . Pathophysiology of gout. Semin Nephrol. 2020;40(6):550‐563. 33678310 10.1016/j.semnephrol.2020.12.001

[2] Fenando A , Rednam M , Gujarathi R , Widrich J . Gout. StatPearls. StatPearls Publishing LLC; 2024. StatPearls PublishingCopyright © 2024. 31536213

[3] Dalbeth N , Gosling AL , Gaffo A , Abhishek A . Gout. Lancet. 2021;397(10287):1843‐1855. 33798500 10.1016/S0140-6736(21)00569-9

[4] Bos MJ , Koudstaal PJ , Hofman A , Witteman JCM , Breteler MMB . Uric acid is a risk factor for myocardial infarction and stroke: the Rotterdam study. Stroke. 2006;37(6):1503‐1507. 16675740 10.1161/01.STR.0000221716.55088.d4

[5] Talpur AS , Fattah A , Hewadmal H , et al. Asymptomatic hyperuricemia as an independent risk factor for myocardial infarction in adult population: a four‐year follow‐up study. Cureus. 2023;15(2):e34614. 36891011 10.7759/cureus.34614 PMC9986685

[6] Mikuls TR , Farrar JT , Bilker WB , Fernandes S , Schumacher HR Jr , Saag KG . Gout epidemiology: results from the UK general practice research database, 1990–1999. Ann Rheum Dis. 2005;64(2):267‐272. 15647434 10.1136/ard.2004.024091 PMC1755343

[7] van Veen TR , Haeri S . Gout in pregnancy: a case report and review of the literature. Gynecol Obstet Investig. 2015;79(4):217‐221. 25660596 10.1159/000369999

[8] Singh JA , Gaffo A . Gout epidemiology and comorbidities. Semin Arthritis Rheum. 2020;50(3, Supplement):S11‐S16. 32620196 10.1016/j.semarthrit.2020.04.008

[9] Capobianco G , Gulotta A , Tupponi G , et al. Materno‐fetal and neonatal complications of diabetes in pregnancy: a retrospective study. J Clin Med. 2020;9(9):2707. 32825775 10.3390/jcm9092707 PMC7564828

[10] Battarbee AN , Sinkey RG , Harper LM , Oparil S , Tita ATN . Chronic hypertension in pregnancy. Am J Obstet Gynecol. 2020;222(6):532‐541. 31715148 10.1016/j.ajog.2019.11.1243

[11] Batt RE , Cirksena WJ , Lebherz TB . Gout and salt‐wasting renal disease during pregnancy; diagnosis, management, and follow‐up. JAMA. 1963;186:835‐838. 14061065 10.1001/jama.1963.03710090035007

[12] Horta‐Baas G , Hernández‐Cabrera MF , Vergara‐Sánchez I , Romero‐Figueroa MS . Gout and pregnancy: a report of a new case and systematic review. Rev Colomb Reumatol. 2017;24(4):219‐229.

[13] Friedman EA , Little WA . Pregnancy and gout: a case report. Am J Obstet Gynecol. 1958;76(4):913‐916. 13583038 10.1016/0002-9378(58)90030-9

[14] Steiner C , Elixhauser A , Schnaier J . The healthcare cost and utilization project: an overview. Eff Clin Pract. 2002;5(3):143‐151. 12088294

[15] Canadian Institutes of Health Research . Natural Sciences and Engineering Research Council of Canada, and Social Sciences and Humanities Research Council. Tri‐Council Policy Statement: Ethical Conduct for Research Involving Humans; 2018.

[16] Chen YK , Wu FJ , Lin HC . Maternal gout associated with increased risk of low birth weight and preterm birth. Int J Gynaecol Obstet. 2010;109(2):157‐158. 20152975 10.1016/j.ijgo.2009.12.016

[17] Powers RW , Bodnar LM , Ness RB , et al. Uric acid concentrations in early pregnancy among preeclamptic women with gestational hyperuricemia at delivery. Am J Obstet Gynecol. 2006;194(1):160.e1‐160.e8. 16389026 10.1016/j.ajog.2005.06.066

[18] Ye W , Luo C , Huang J , Li C , Liu Z , Liu F . Gestational diabetes mellitus and adverse pregnancy outcomes: systematic review and meta‐analysis. BMJ. 2022;377:e067946. 35613728 10.1136/bmj-2021-067946 PMC9131781

[19] Egan AM , Dow ML , Vella A . A review of the pathophysiology and management of diabetes in pregnancy. Mayo Clin Proc. 2020;95(12):2734‐2746. 32736942 10.1016/j.mayocp.2020.02.019

[20] Seely EW , Ecker J . Chronic hypertension in pregnancy. Circulation. 2014;129(11):1254‐1261. 24637432 10.1161/CIRCULATIONAHA.113.003904

[21] Attali E , Yogev Y . The impact of advanced maternal age on pregnancy outcome. Best Pract Res Clin Obstet Gynaecol. 2021;70:2‐9. 32773291 10.1016/j.bpobgyn.2020.06.006

[22] Choi HK , Ford ES . Haemoglobin A1c, fasting glucose, serum C‐peptide and insulin resistance in relation to serum uric acid levels—the third National Health and nutrition examination survey. Rheumatology. 2008;47(5):713‐717. 18390895 10.1093/rheumatology/ken066

[23] Facchini F , Chen YD , Hollenbeck CB , Reaven GM . Relationship between resistance to insulin‐mediated glucose uptake, urinary uric acid clearance, and plasma uric acid concentration. JAMA. 1991;266(21):3008‐3011. 1820474

[24] Kubota Y , McAdams‐DeMarco M , Folsom AR . Serum uric acid, gout, and venous thromboembolism: the atherosclerosis risk in communities study. Thromb Res. 2016;144:144‐148. 27337701 10.1016/j.thromres.2016.06.020 PMC5120547

[25] Chiu CC , Chen YT , Hsu CY , et al. Association between previous history of gout attack and risk of deep vein thrombosis—a nationwide population‐based cohort study. Sci Rep. 2016;6:26541. 27231197 10.1038/srep26541 PMC4882589

[26] Țăpoi L , Șalaru DL , Sascău R , Stătescu C . Uric acid‐an emergent risk marker for thrombosis? J Clin Med. 2021;10(10):2062. 34065792 10.3390/jcm10102062 PMC8150596

